# Introduction

**DOI:** 10.1155/S146392468300019X

**Published:** 1983

**Authors:** R. Haeckel

**Affiliations:** Institut für Klinische Chemie Medizinische Hochschule Hannover Germany


Journal of Automatic Chemistry, Volume 5, Number 2 (April–June 1983), pages 68–70

### Editorial

### Introduction to the papers presented at the symposium on cost analysis held on 22 April 1982 in Barcelona during the First International Congress on Automation in Clinical Chemistry

Cost analysis was accepted at the Barcelona conference as a special topic—this is the first time an international meeting has included the subject. The two major areas where cost analysis is urgently required in a medical laboratory which has an increasing lack of funds are in the discussion of strategies for selecting tests (which test, for example, has the highest discriminative power for a special diagnostic problem?), and in the choice of instruments (which instrument, or analytical system, produces the least costs for special diagnostic strategies?). Cost analysis is becoming more and more important to decision-making in medical laboratories.

Although the need for cost analysis appears to be generally accepted, it is still undecided as to who should collect and allocate costs and how this should be achieved. Comparison of costs can only be useful if the processes of collection and allocation are standardized and if definitions of terms used are internationally accepted—the language applied must be understood by all laboratory personnel.

The purpose of the symposium on cost analysis at Barcelona was to provide a general summary of the present status of cost analysis in the medical laboratory. Because of a limit on time, the reports presented could only introduce the field of costing. And the symposium was intended to stimulate interest in costing rather than to provide a detailed overview of the field.

The relation of the cost analysis symposium to automation (the primary topic of the Barcelona Congress) was evident: economical viewpoints are becoming more important, especially in terms of expensive instrumentation and large, automated multi-test systems which now influence the whole infrastructure of a medical laboratory.

The papers read at the symposium follow.

*Editor's note*: one symposium paper, received too late for this issue, will be published in *Journal of Automatic Chemistry*, Vol. 5, No. 3.

### Introduction

### Cost-benefit analysis

**R. Haeckel**

*Institut für Klinische Chemie, Medizinische Hochschule, Hannover, FR Germany*

The decision-making process surrounding the purchase of new instruments in a medical laboratory involves several steps, and many aspects have to be considered; this point was discussed during the cost-analysis symposium organized by the IFCC's Expert Panel on Instrumentation in collaboration with the IUPAC Commission on Automation and held at 1982's Barcelona meeting. The purpose of this paper is to discuss the economic aspects of the process. Because large analytical systems influence the whole organizational structure of the laboratory, cost analysis and cost-benefit analysis must be considered in respect of the total laboratory structure. The primary task of the medical laboratory is to produce test results—these usually lead to a number of effects:

Test
↓←Expenditure
Result– – →Efficiency
↓
Effect(s)– – →Effectiveness

The laboratory's efficiency in fulfilling this task can be measured by the quantity of results and by the costs incurred in obtaining them (expenditure). The laboratory's effectiveness is judged by its 'utility'. ('Utility' is a term commonly used in epidemiology and in social economics—see references [1–8].)

Costs are usually considered in monetary units, whereas utility can be monitored in monetary and in non-monetary units. Before costs are discussed in detail a few aspects of utility need to be considered.

Generally, an effective diagnostic strategy should lead to a gain in utility. The expense must, in some way, serve the patient, even if this is only a reduction in uncertainty [5]. The utility of diagnostic and therapeutic steps can be a reduction of disease course, a gain in life expectancy or prognostic hints. Utility can be studied on a micro- or macroeconomic scale. In the first case, utility is considered in a small unit (for example when buying a new instrument); in the second, utility is looked at on a much larger scale—its impact on a population for instance. If a negative test result leads to an unjustified therapy being followed or to an extension of a disease course, a loss of utility results. 'Net utility' is the sum of all positive and negative utilities.

Cost-utility investigations have been divided into cost-benefit and cost-effectiveness analyses [1–8] (see table 1).

In a cost-benefit analysis, expenses and utilities are considered in monetary units, these are finally summed so that the result is an amount of money. In a cost-effectiveness analysis, however, costs are collected in monetary units and utilities in other units: these units may be years of life expectancy, days of treatment, morbidity and mortality rates etc. The results are expressed in, for instance, monetary units per number of persons treated successfully.

The transformation of primarily non-monetary units into monetary equivalents is difficult [2–9] and will not be discussed here. Therefore, on one side all monetary gains and losses are summed and on the other side are all the non-monetary positive and negative utilities. The result of such an analysis is net costs per net utilities.

*Table 1. Cost-utility analyses.*

| Name of analysis | Cost-benefit analysis | Cost-effectiveness analysis |
|---|---|---|
| Result of analysis | Immediate costs per number of tests | Total costs per utility |
| Result of analysis is an indicator for | Efficiency | Effectiveness |

The total expenses are often difficult to quantify, even if only single tests have to be considered. A diagnostic strategy with several simultaneously and/or sequentially performed tests leads to an even more complicated cost analysis.

The total costs of a test ($C_{tot}$) consist of the immediate costs ($C_t$) (caused by performing the test) and induced costs ($C_i$) (these are related to the effects caused by the test). If a diagnostic-therapeutic strategy is based on binary decisions, then the expected induced costs (*a priori* induced costs) can be calculated according to the formula:

$$\Delta C = p \cdot C$$

($p$ represents probability and $C$ costs per event). The following induced costs can be relevant to a cost-effectiveness analysis [10]:

(1) Costs for further diagnostic procedures ($\Delta C_{fd}$).
(2) Costs for therapeutic actions taken because of positive tests (including false positive results) ($\Delta C_t$).
(3) Costs incurred preventing morbidity ($\Delta C_m$).
(4) Costs resulting from side-effects of the diagnostic therapeutic strategy ($\Delta C_n$).
(5) Costs incurred from diseases resulting from a prolonged life ($\Delta C_1$).
(6) Costs caused by omitting a necessary therapy as a consequence of false negative results ($\Delta C_{fn}$).

Summing these single costs leads to the induced costs $C_i$:

$$C_i = \Delta C_{fd} + \Delta C_t - \Delta C_m + \Delta C_n + \Delta C_1 + \Delta C_{fn}.$$

So the cost-effectiveness equation can now be written as:

$$\frac{C_{tot}}{U_{tot}} = \frac{\text{total net costs}}{\text{total net utilities}}.$$

Whilst induced costs of a test are currently very difficult to quantify, many laboratories have now started to record immediate costs: this is known as 'cost accounting'.

Calculation of a test's costs requires collection of all the costs involved in performing a particular test. In some cases, however, it is unnecessary to conduct such a comprehensive cost analysis. For the purpose of investment decisions it is sometimes sufficient to consider only a part of all costs. Such a problem-oriented cost analysis will be discussed later.

Before collecting details of costs involved in a test, it needs to be decided which costs are relevant. Usually, costs are divided into various kinds of costs. A criterion for this differentiation can be the method of distribution: costs can be allocated directly or indirectly [11 and 12].

Direct costs occur in relation to the test requested at the principal cost centres, which usually correspond with the work stations where the particular test is performed. Direct costs can be attributed directly to the test (see table 2): they are either fixed, semi-variable or variable. Fixed costs are time-related (figure 1), but they are independent of the number of tests, as for example the purchase cost of an instrument. Variable costs depend on the number of tests: costs of reagents and disposables. Semi-variable costs increase with the number of tests performed, usually this is done at intervals—for example when the batch size has reached a particular length. Staff costs can be either variable or semivariable.

Indirect costs (see table 3) occur at overhead cost centres, for example internal administration office or workshop. They have to be distributed among the main cost centres.

In table 4 the most important test costs are listed. They contain all the analytical and post-analytical, but not the pre-analytical, costs. Pre-analytical costs concern preparation of the patient, venipunctures and specimen transport.

A complete internal cost accounting, which covers all kinds of costs and performs all the necessary allocation steps can only be done, acceptably in terms of expense, if electronic data-processing is available.

For assisting investment decisions, problem-oriented cost analysis is often sufficient and requires only some of the costs. In most investment decisions, a limited number of alternatives have

*Table 2. Direct costs.*

| | |
|---|---|
| 1. | *Fixed costs* |
| 1.1 | Costs of laboratory space |
| 1.2 | Depreciation of instruments |
| 1.3 | Interests |
| 1.4 | Maintenance and servicing |
| 2. | *Semi-variable costs* |
| 2.1 | Costs of personnel |
| 2.2 | Costs of quality control |
| 3. | *Variable costs* |
| 3.1 | Costs of reagents and purified water |
| 3.2 | Costs of disposables |
| 3.3 | Costs of power |
| 3.4 | Costs of repair |

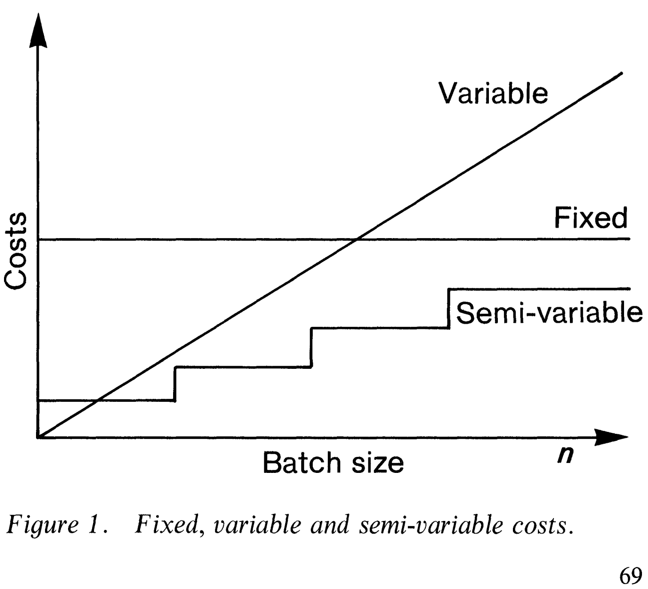

*Figure 1. Fixed, variable and semi-variable costs.*

*Table 3. Indirect costs.*

1. *Overheads inside the laboratory*
1.1 Administration, management, postage, telephone charges, insurance etc.
1.2 Data-processing
1.3 Cleaning
1.4 Cost distribution for staff rooms, floors etc.
1.5 Depreciation for basic instrumentation, furniture etc.

2. *Overheads from outside the laboratory*
2.1 Costs from other central institutions which perform services for the laboratory
2.2 Share in the cost for central administration and operating of the hospital

*Table 4. Total costs of a bearer of costs (for example determination of the glucose concentration, per year).*

1. *Variable costs*
1.1 Reagents (including control materials)
1.2 Disposables
1.3 Costs of personnel
1.4 Power
1.5 Variable overhead costs of the cost centre
1.6 Overhead for central specimen distribution
1.7 Overhead for internal administration
1.8 Overhead for data-processing

2. *Fixed costs*
2.1 Depreciation
2.2 Interest charges
2.3 Service and maintenance
2.4 Fixed overhead costs of the cost centre
2.5 Quality control

3. *Sum of variable and fixed costs*
(1) Effective work time times cost for effective work per minute
(2) Allocated in per cent of the number of tests requested

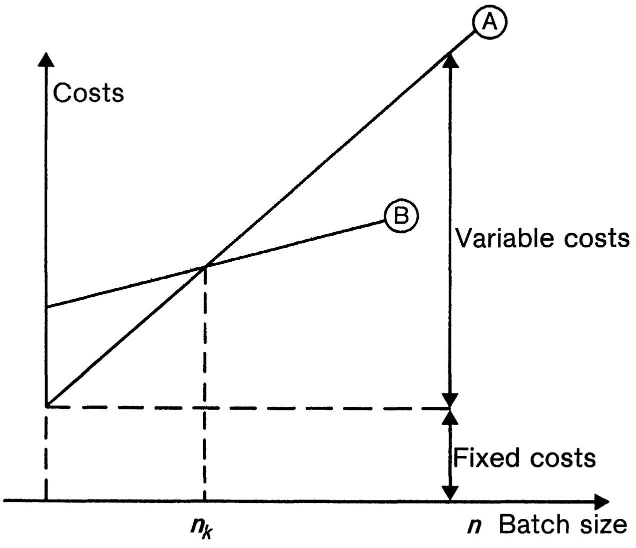

*Figure 2. The critical batch size ($n_c$) as break-even-point. 'A' = (manual) procedure with lower mechanization than 'B' (for example an automated procedure).*

*Table 5. Simplified calculation of the critical batch size [14 and 15].*

$$n_c = \frac{(C_{fB}-C_{fA})+P\cdot 1{,}11(t_{B1}-t_{A1})+P\cdot 0{,}11(t_{A2}-t_{B2})}{(R_{tA}-R_{tB})+(D_{tA}-D_{tB})+P\cdot 0{,}01(t_{A2}-t_{A1}-t_{B2}+t_{B1})}$$

$C_f$ = Fixed costs

$R_t$ = Reagent costs per test

$D_t$ = Costs of disposables per test

$t$ = Technician time for $n_1$ specimen ($t_1$) and for $n_2$ specimen ($t_2$)

$A, B$ = Procedure A (manual or less mechanized) and B (more mechanized or automated)

to be compared with each other. Only those direct costs in which relevant differences are expected need to be considered. The costs for the alternative investments are listed (see, for example, table 4), and the sums are compared with each other: this is a simple cost-benefit analysis. The expenses for an investment are the costs in monetary units, the effect is in terms of, for example, a reduction in personnel time. Since personnel time can be transformed into monetary units, a cost-benefit analysis as demonstrated of micro-economical area [1], is both useful and fairly common.

Another procedure calculates the break-even point at which two alternative solutions produce the same total costs (figure 2). This point has also been called the critical batch size [14 and 15]. It defines the average number of tests which have to be performed in a daily batch in order to cost the same amount of money for two alternatives. If fewer tests have to be performed then solution A is cheaper; if more tests, as stated by the critical batch size, have to be performed then solution B is the more economical. It has been shown that only five data are required to calculate the critical batch size (see table 5).

After this general introduction to the area of laboratory cost analysis, the following papers will cover more specific topics.

### References

1. Henke, K. D. In *Sozialmedizinische Probleme der Hypertonie in der Bundesrepublik Deutschland*, Ed. K. D. Bock (G. Thieme Verlag, Stuttgart, 1978), p. 42.
2. Card, W. I. and Emerson, P. A., *British Medical Journal*, **281** (1980), 543.
3. Galen, R. S., *Arch. Path. Lab. Med.*, **101** (1977), 561.
4. Partington, M. W., *Canadian Medical Association Journal*, **99** (1968), 644.
5. Lindley, D. V. In *Decision Making and Medical Care*, Eds. F. T. de Dombal and F. Gremy (North Holland Publishing Co., 1976), p. 101.
6. Schweitzer, St. O., *Health Services Research* (1974), 22.
7. Card, W. I. and Mooney, G. H., *British Medical Journal*, **2** (1977), 1627.
8. Brünggger, H., *Die Nutzen-Kosten-Analyse als Instrument der Planung im Gesundheitswesen* (Schulthess Polygraphischer Verlag, Zurich, 1974), p. 1.
9. Mooney, G. H., *The Valuation of Human Life* (The Macmillan Press Ltd, London & Basingstoke, 1977).
10. Weinstein, M. C. and Feinberg, H. V. In *Logic and Economies of Clinical Laboratory Use*, Eds. E. S. Benson and M. Rubin (1977).
11. Lundberg, G. D. and Westlake, G. E., *Journal of the American Medical Association*, **243** (1980), 1659.
12. Broughton, P. M. G. and Hogan, T. C., *Am. Clin. Biochem.*, **18** (1982), 330.
13. Haeckel, R. and Weirich, A., *GIT Lab. Med.*, **5** (1982), 199.
14. Haeckel, R., Höpfel, P. and Höner, G., *Journal of Clinical Chemistry and Clinical Biochemistry*, **12** (1974), 14.
15. Haeckel, R., *Medical Progress Through Technology*, **3** (1975), 65.

